# Local Delivery of Streptomycin in Microcontainers Facilitates Colonization of Streptomycin-Resistant Escherichia coli in the Rat Colon

**DOI:** 10.1128/aem.00734-22

**Published:** 2022-06-27

**Authors:** Anders M. Torp, Khorshid Kamguyan, Juliane F. Christfort, Katja Ann Kristensen, Priscila Guerra, Noëmie Daniel, Line Hagner Nielsen, Kinga Zòr, Benoit Chassaing, Anja Boisen, Martin I. Bahl, Tine Rask Licht

**Affiliations:** a The National Food Institute, Technical University of Denmark, Kongens Lyngby, Denmark; b Department of Health Technology, Technical University of Denmark, Kongens Lyngby, Denmark; c INSERM U1016, Team “Mucosal Microbiota in Chronic Inflammatory Diseases,” CNRS UMR 8104, Université de Paris, Paris, France; University of Naples Federico II

**Keywords:** Gut microbiota, streptomycin, *Escherichia coli*, colonization, local delivery, 16S rRNA amplicon sequencing, microbiome analysis, laser capture microdissection, microcontainers, rats, gut microbiome, intestinal colonization

## Abstract

Oral antibiotic treatment is often applied in animal studies in order to allow establishment of an introduced antibiotic-resistant bacterium in the gut. Here, we compared the application of streptomycin dosed orally in microcontainers to dosage through drinking water. The selective effect on a resistant bacterial strain, as well as the effects on fecal, luminal, and mucosal microbiota composition, were investigated. Three groups of rats (*n* = 10 per group) were orally dosed with microcontainers daily for 3 days. One of these groups (STR-M) received streptomycin-loaded microcontainers designed for release in the distal ileum, while the other two groups (controls [CTR] and STR-W) received empty microcontainers. The STR-W group was additionally dosed with streptomycin through the drinking water. A streptomycin-resistant Escherichia coli strain was orally inoculated into all animals. Three days after inoculation, the resistant E. coli was found only in the cecum and colon of animals receiving streptomycin in microcontainers but in all intestinal compartments of animals receiving streptomycin in the drinking water. 16S rRNA amplicon sequencing revealed significant changes in the fecal microbiota of both groups of streptomycin-treated animals. Investigation of the inner colonic mucus layer by confocal laser scanning microscopy and laser capture microdissection revealed no significant effect of streptomycin treatment on the mucus-inhabiting microbiota or on E. coli encroachment into the inner mucus. Streptomycin-loaded microcontainers thus enhanced proliferation of an introduced streptomycin-resistant E. coli in the cecum and colon without affecting the small intestine environment. While improvements of the drug delivery system are needed to facilitate optimal local concentration and release of streptomycin, the application of microcontainers provides new prospects for antibiotic treatment.

**IMPORTANCE** Delivery of antibiotics in microcontainer devices designed for release at specific sites of the gut represents a novel approach which might reduce the amount of antibiotic needed to obtain a local selective effect. We propose that the application of microcontainers may have the potential to open novel opportunities for antibiotic treatment of humans and animals with fewer side effects on nontarget bacterial populations. In the current study, we therefore elucidated the effects of streptomycin, delivered in microcontainers coated with pH-sensitive lids, on the selective effect on a resistant bacterium, as well as on the surrounding intestinal microbiota in rats.

## INTRODUCTION

The gut microbiota plays a pivotal role in immune system homeostasis and health of the host (1). One of the main functions of the gut microbiota is to prevent the colonization of invading microbes through a well-described phenomenon designated colonization resistance, which is promoted by microbe-microbe and microbe-host interactions (2). Investigation of specific bacterial functions in the gut is often explored in animals by oral introduction of bacteria. Antibiotic treatment may in this context be applied to reduce the colonization barrier of the microbiota and allow proliferation of inoculated bacteria resistant to the antibiotic (3, 4). The antibiotic is typically added to the drinking water and, thereby, distributed through the gastrointestinal tract until absorbed or secreted. However, a delayed and more localized release, targeting specific bacterial spatial niches, may be obtained by encapsulating the orally administered compound in specifically designed delivery vehicles. Novel polymeric delivery vehicles, such as micrometer-sized microspheres or microdevices, are more adaptable than conventional capsule delivery devices (5, 6). One promising type of microdevices is microcontainers, a spherical polymeric reservoir with an opening at the top. Previous studies have demonstrated the efficacy of microcontainers to protect the loaded content in various *in vitro* and *in vivo* conditions (7). After loading, the microcontainer is coated with a pH- or enzyme-sensitive coating (8) to control the speed and location of the release. Microcontainers have been used to study oral delivery of amoxicillin and to increase systemic uptake of this drug in the intestines of rats (9). Microcontainers have also been seen to improve bioavailability of the drugs furosemide (10) and ketoprofen (11) *in vivo*.

**FIG 1:**
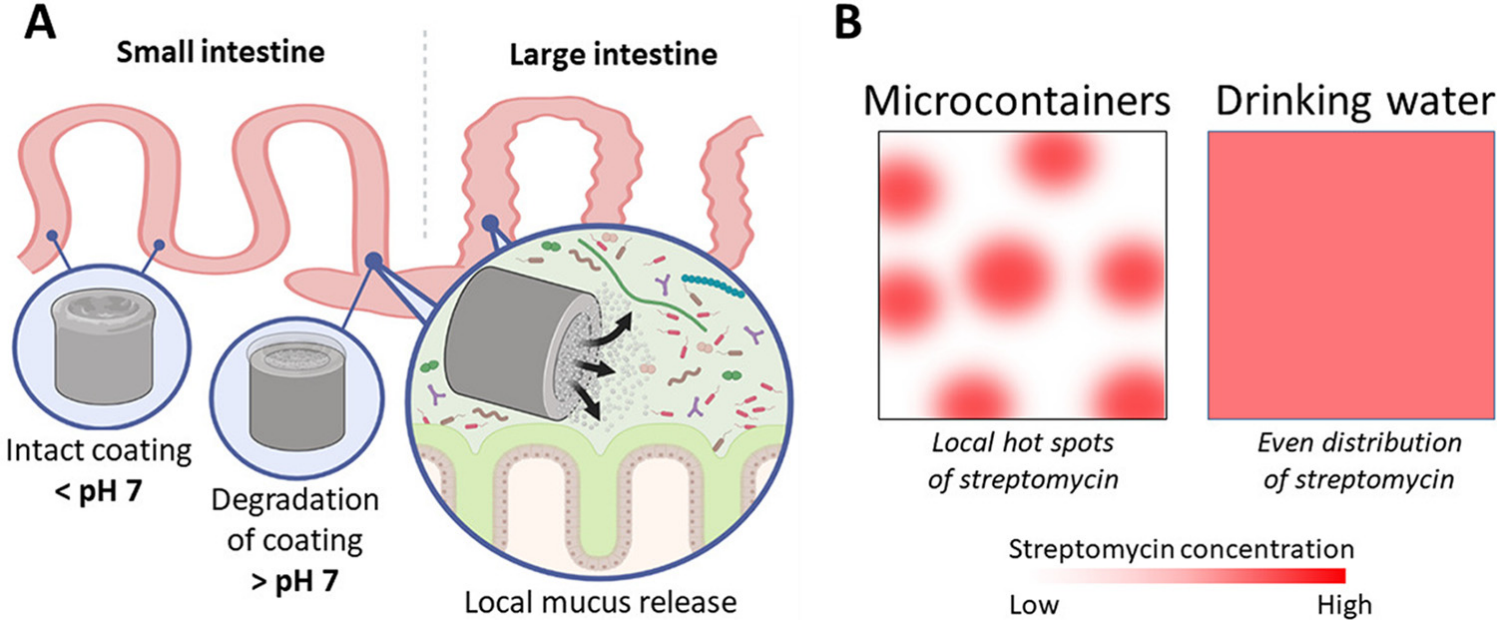
Illustration of local microcontainer-mediated streptomycin delivery and distribution in the intestinal environment. (A) Streptomycin is confined inside the microcontainers until the pH-sensitive coating is degraded at approximately pH 7. (B) Anticipated distribution of streptomycin in the intestinal environment, resulting in local hot spots when delivered by microcontainers and in a more even distribution when administered in drinking water. (Illustration created with BioRender).

In this study, we tested the feasibility of microcontainers (Fig. 1) for the delivery of streptomycin, which is poorly absorbed, to the colon of rats and evaluated the effect of this local delivery on the selective proliferation of an introduced streptomycin-resistant (Str^r^) strain of Escherichia coli. Additionally, the impact of microcontainer-based delivery of streptomycin on the intestinal luminal and mucosal microbiotas was assessed by 16S rRNA amplicon sequencing and compared to the effect obtained by delivery of 5 g/L streptomycin in drinking water, which is the current standard for rodent experimentation requiring bacterial colonization (12, 13).

## RESULTS

### Streptomycin delivered in microcontainers allows colonization of streptomycin-resistant E. coli.

**FIG 2:**
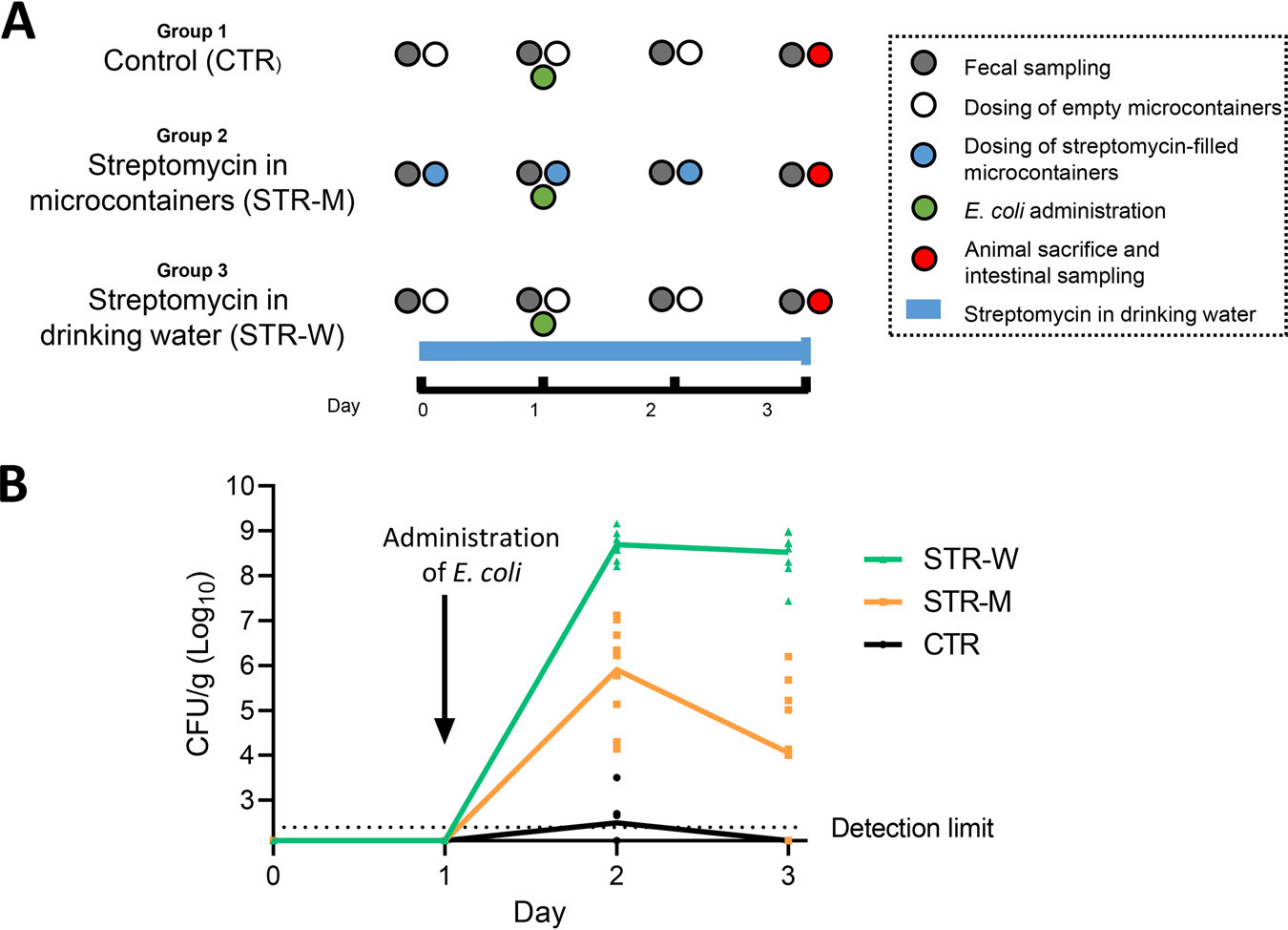
Schematic representation of the *in vivo* study (A) and detection of Str^r^ E. coli in feces (B). Significantly different loads of Str^r^ E. coli were observed between all groups (*P* < 0.01, multiple unpaired *t* tests) on day 2 and day 3 as well as for STR-M between day 2 and 3 (*P* < 0.01, paired *t* test), assessed by CFU counting. Detection limit, 250 CFU/g feces; values below the detection limit were set to half detection.

Fecal samples were obtained throughout the study (Fig. 2A), and no Str^r^ E. coli was detected in any of the groups before administration of this strain (Fig. 2B). One day after administration (day 2), the average numbers of Str^r^ E. coli were significantly increased in groups receiving streptomycin in microcontainers (STR-M) or drinking water (STR-W), but not in the control (CTR) group. Between 24 and 48 h after E. coli administration, counts decreased from 5.91 ± 1.06 log CFU/g to 4.06 ± 1.53 log CFU/g in the STR-M group, whereas counts in the STR-W group were unchanged, going from 8.69 ± 0.31 log CFU/g to 8.52 ± 0.46 log CFU/g. No E. coli was detected in the control group 48 h after inoculation, indicating that the bacterium was not able to proliferate in the animals without streptomycin treatment. A larger variation was observed in the counts obtained in the STR-M group than in the STR-W group, probably reflecting the difference between a distinct once-a-day treatment with streptomycin versus a more continuous ingestion with *ad hoc* drinking water.

### Streptomycin-resistant E. coli colonizes the cecum and colon of rats receiving streptomycin in microcontainers.

**FIG 3:**
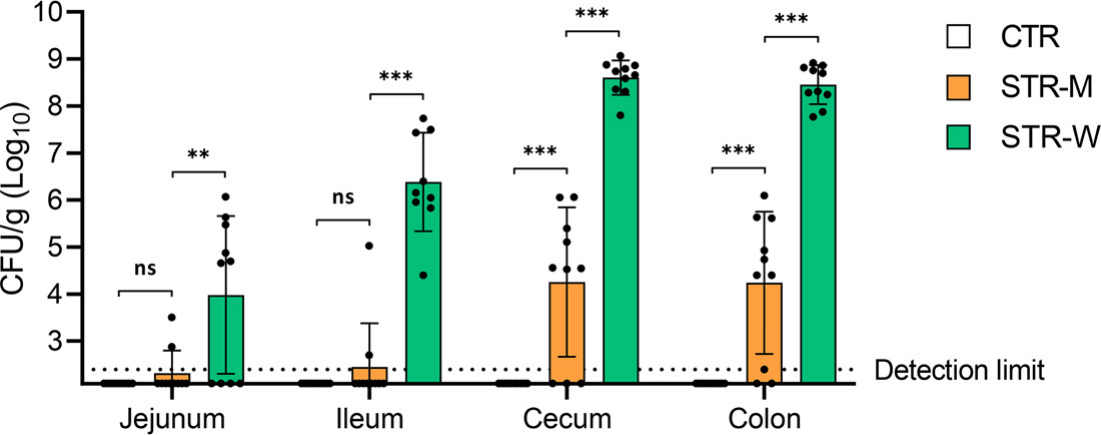
Detection of Str^r^ E. coli in gut compartments 2 days after administration. Delivery of streptomycin in microcontainers targeting the large intestine allowed selective proliferation of Str^r^ E. coli in the cecum and colon, but not in the small intestine, as opposed to streptomycin delivery in the drinking water, which allowed significantly higher loads of Str^r^ E. coli in all intestinal compartments. Note that the total amount of streptomycin delivered by microcontainers in STR-M animals was lower than the total amount delivered by drinking water in STR-W animals, which likely explains the cecal and colonic differences between the counts in these two groups. Detection limit, 250 CFU/g feces; values below the detection limit are set to half detection. *, *P* < 0.05; **, *P* < 0.01; ***, *P* < 0.001; ns, not significant.

In line with the observation from fecal samples, Str^r^ E. coli was not detectable in any of the sampled intestinal compartments (jejunum, ileum, cecum, and colon) of the CTR group on day 3 (Fig. 3), confirming that the introduced bacteria were rapidly shed from the gut of non-streptomycin-treated animals. In 2 of the 10 STR-M animals, E. coli was detectable in the jejunum and ileum, indicating premature release of streptomycin from the microcontainers in the small intestine. Nevertheless, the average E. coli counts in the small intestinal sections of the STR-M group were not significantly different from those in the CTR group. In contrast, significantly higher numbers of Str^r^ E. coli were found in the jejunum and ileum from the STR-W group than from the CTR group, reaching 3.98 ± 1.68 log CFU/g and 6.38 ± 1.05 log CFU/g, respectively.

In the cecum and colon, both of the streptomycin-treated groups (STR-M and STR-W) contained significantly higher numbers of E. coli than the CTR. In the STR-M group, 4.26 ± 1.59 and 4.22 ± 1.51 log CFU/g were measured in the cecum and colon, respectively. In the STR-W group, 10^4^-fold-higher counts of 8.6 ± 0.37 log CFU/g in the cecum and 8.45 ± 0.42 log CFU/g in the colon were detected. In three of the STR-M animals, E. coli was undetectable in all intestinal compartments (Fig. 3) as well as in feces (Fig. 2B), suggesting that release of streptomycin from the microcontainers had failed in these animals.

### A low dose of streptomycin in microcontainers impacts intestinal microbiota composition.

**FIG 4:**
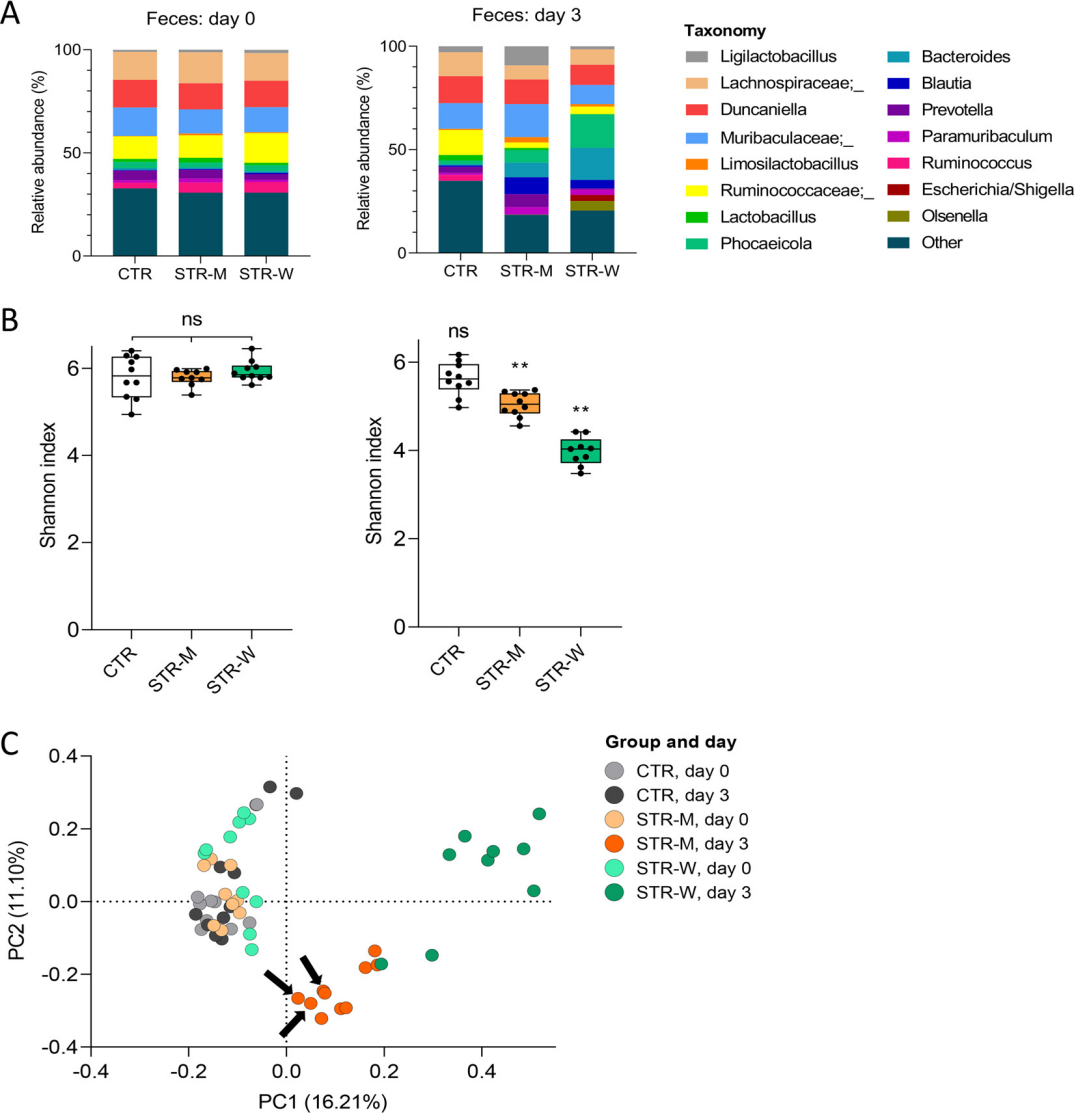
Changes in fecal microbiota after streptomycin treatments for 3 days. (A) Bar plots of the bacterial genus distribution in feces before and after 3 days of streptomycin treatment. A few groups were only identified at the family level. (B) Box plots of the fecal microbiota alpha diversities on day 0 (left) and day 3 (right). Statistics shown in the day 3 plot are based on comparisons to the same group at day 0. *, *P* < 0.05; **, *P* < 0.01; ***, *P* < 0.001. (C) PCoA plots of Bray-Curtis dissimilarities of the fecal microbiota. Arrows in the PCoA plot denote samples from the three animals showing no Str^r^ E. coli detected. Significant differences were found between CTR and STR-W animals (*P* = 0.01, ANOSIM) on day 0 and for STR-M (*P* < 0.01, ANOSIM) and STR-W (*P* < 0.01, ANOSIM) between day 0 and day 3. No significant differences were found between CTR and STR-M (*P* = 0.15, ANOSIM) or STR-M and STR-W (*P* = 0.09, ANOSIM) on day 0 or between day 0 and day 3 for CTR (*P* = 0.53, ANOSIM).

Animals receiving streptomycin in microcontainers were each fed one microcontainer-filled capsule a day, equivalent to about 3.19 mg of streptomycin, whereas the animals receiving streptomycin in the drinking water were estimated to have ingested around 250 mg of drug per day. To assess the effect on the gut microbiota of a low dose of streptomycin delivered in microcontainers, fecal and luminal gut contents were profiled by 16S rRNA amplicon sequencing (Fig. 4A). Within-sample diversity, i.e., alpha diversity, was assessed by the Shannon index, whereas between-sample diversity, i.e., beta diversity, was assessed by principal component analysis (PCoA) of Bray-Curtis dissimilarities. While there were no differences in the alpha diversity between the three groups at day 0 (*P* = 0.49, Kruskal-Wallis test) (Fig. 4B), a small but significant difference in beta diversity at day 0 was observed between the CTR and STR-W groups (*P* = 0.01, *R* = 0.19, analysis of similarities [ANOSIM], Bray-Curtis) (Fig. 4C). Before streptomycin administration (day 0), no differences in fecal beta diversity were observed between the CTR and STR-M groups (*P* = 0.15, *R* = 0.08, ANOSIM, Bray-Curtis) (Fig. 4C) nor between the STR-M and STR-W groups (*P* = 0.09, *R* = 0.11 ANOSIM, Bray-Curtis) (Fig. 4C). The fecal microbiota changed between day 0 and day 3 in both streptomycin-treated groups (for both, *P* < 0.01, ANOSIM, Bray-Curtis) (Fig. 4C), accompanied by a significant decrease in alpha diversities (for both, *P* < 0.01, Wilcoxon) (Fig. 4B). A PCoA based on Bray-Curtis dissimilarities (Fig. 4C) revealed distinct clustering of the streptomycin-treated groups at day 3. The gut microbiota did not change significantly in the CTR group between day 0 and day 3 (*P* = 0.53, ANOSIM, Bray-Curtis), indicating that the presence of microcontainers without streptomycin content did not affect the gut microbiota.

Before streptomycin treatment, the relative abundance of Escherichia and *Shigella* in the fecal samples as assessed by 16S rRNA gene profiling was below 0.01% in all groups. The relative abundance of Escherichia and *Shigella* increased to 3.00% (±1.95% standard deviation [SD]) after streptomycin treatment in the STR-W group, but no increases in the other groups were detected, suggesting that the 16S-based profiling did not capture the increase of E. coli detected by the more sensitive CFU enumeration in the STR-M group. The Escherichia and *Shigella* relative abundance assessed by 16S rRNA profiling showed a positive correlation to the Str^r^ E. coli CFU counts from fecal and intestinal samples (*R*^2^ = 0.67), suggesting that the relative abundance changes were primarily caused by colonization of the introduced E. coli and did not reflect changes in endogenous E. coli populations of the animals (see Fig. S1 in the supplemental material).

### Microcontainer-delivered streptomycin affects the cecal and colonic microbiota but not the small intestinal microbiota.

**FIG 5:**
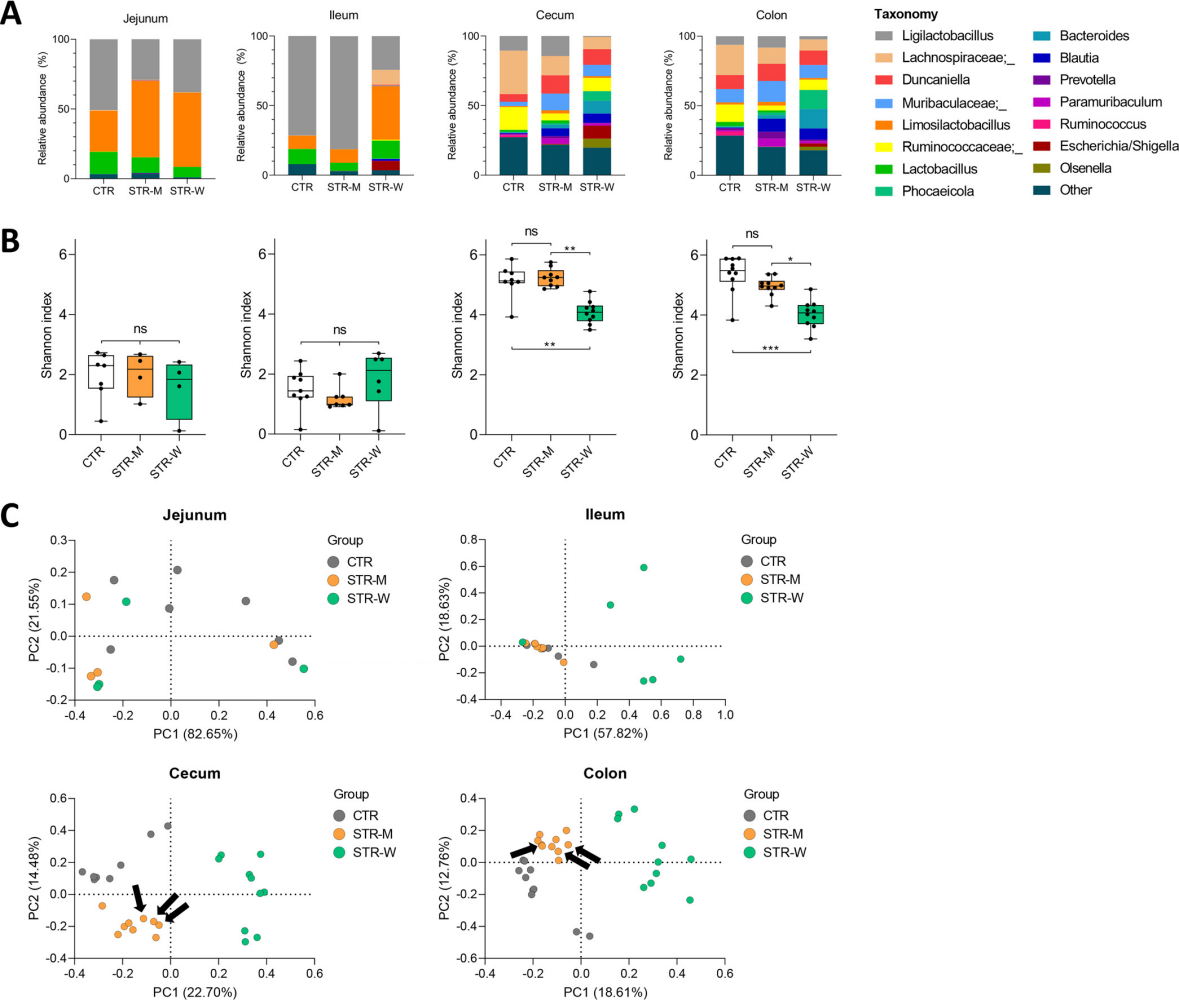
Changes in the microbiota in different gut compartments following streptomycin administration for 3 days. (A) Bar plots of the bacterial taxonomy distribution in gut compartments after 3 days of streptomycin treatment. (B) Box plots of the gut microbiota alpha diversities. *, *P* < 0.05; **, *P* < 0.01; ***, *P* < 0.001. (C) PCoA plots of the gut microbiota based on Bray-Curtis dissimilarities. Arrows in the PCoA plot mark communities from the three animals showing no Str^r^ E. coli detection. No significant differences were found between the three groups in jejunum (CTR versus STR-M, *P* = 0.23; CTR versus STR-W, *P* = 0.30; STR-M versus STR-W, *P* = 0.70, ANOSIM). In ileum, a significant difference was found between CTR and STR-W groups and the STR-M and STR-W groups (CTR versus STR-W, *P* < 0.01; STR-M versus STR-W, *P* < 0.01, ANOSIM) but not between CTR and STR-M (*P* = 0.47, ANOSIM). In the cecum and colon, significant differences were found between all groups (all comparisons, *P* < 0.01, ANOSIM).

Fecal and intestinal samples were analyzed by 16S ribosomal gene amplicon sequencing (Fig. 5A). No differences in alpha diversity were observed between treatment groups in the jejunal and ileal compartments (Fig. 5B). The relative abundance of Escherichia and *Shigella* in the jejunum was <0.01% in all three groups. Similarly, the jejunal and ileal beta diversities did not differ between the CTR and STR-M groups (Fig. 5C). However, the STR-W group displayed a significantly different ileal community from the two other groups (Fig. 5C). In the ileum, the relative abundance of Escherichia and *Shigella* was 6.72% (±7.87%) in the STR-W group, while it was <0.001% in both the STR-M and CTR groups.

In the cecum and colon, significant differences in beta diversity were observed between the two streptomycin treatment groups and the CTR group (for both comparisons, *P* ≤ 0.001, ANOSIM, Bray-Curtis) (Fig. 5C), while the alpha diversity was significantly lowered only in the STR-W group (Fig. 5B). The relative abundances of Escherichia and *Shigella* remained <0.01% in cecum and colon of the control animals. In the STR-M and STR-W groups, the relative abundances were 0.48% (±0.86%) and 9.40% (±5.56%) in the cecum and <0.01% and 2.42% (±1.32%) in the colon, respectively, in line with the CFU enumerations (Fig. 3).

### Streptomycin does not increase the colonization of E. coli in the inner colonic mucus layer.

**FIG 6:**
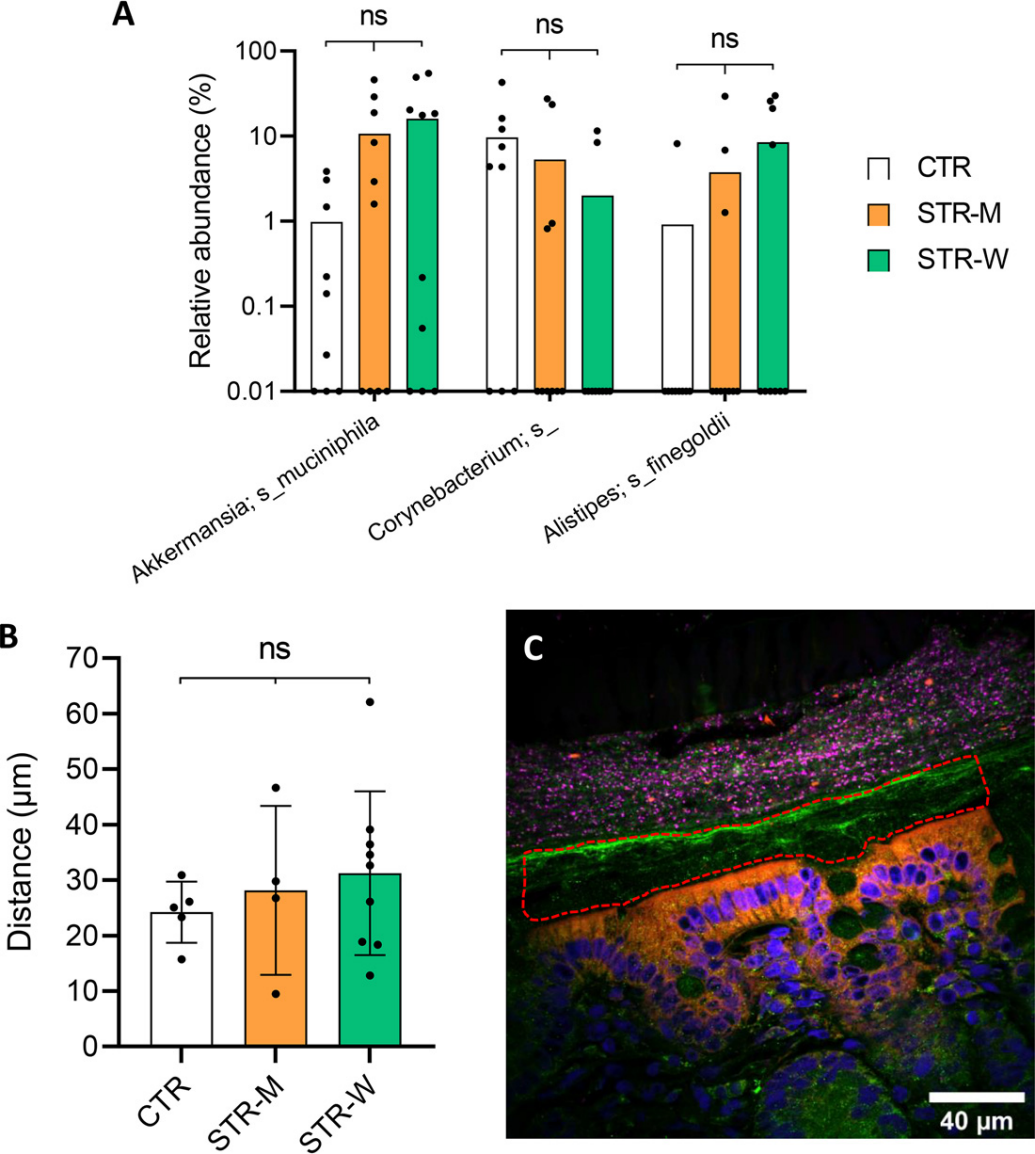
Analysis of the inner colonic mucus layer. (A) Relative abundances of the three most prevalent bacteria found in the colonic inner mucus layer. No statistically significant differences were found for *A. muciniphila* (*P* = 0.47, one-way ANOVA), *Corynebacterium* sp. (*P* = 0.11, one-way ANOVA), or *A. finegoldii* (*P* = 0.32, one-way ANOVA) between the three groups. Undetected bacteria were set to a relative abundance of 0.01%. (B) Distances from the intestinal epithelium cells (IEC) to the microbiota were not significantly altered by the streptomycin treatments (*P* = 0.64, one-way ANOVA). (C) Representative confocal laser scanning microscopy (CLSM) image of the colonic mucosa with epithelial cells (blue, DNA), epithelium border (orange, actin), mucus (green. MUC2), and the gut bacteria (purple, 16S gene). The dotted red line represents a typical sample collected by laser capture microdissection.

16S rRNA gene sequencing was applied on laser capture microdissected inner mucus layer, in order to see whether release of streptomycin from microcontainers embedded in the mucus might affect encroachment of E. coli or modulate the sparse bacterial population interacting directly with the epithelium. Escherichia was not detected in the mucosal compartments in any of the three groups. The three most abundant bacterial species found in the inner mucus were Akkermansia muciniphila, a *Corynebacterium* sp., and Alistipes finegoldii (Fig. 6A). Even though the higher relative abundance of *A. muciniphila* observed in the mucus of STR-W animals was not statistically significant (*P* = 0.23, unpaired *t* test), it is worth noting that *A. muciniphila* relative abundance was generally lower in the CTR group. No significant changes were found for the *Corynebacterium* sp. and Alistipes finegoldii between the groups. Additionally, no difference in the distance between the microbiota and the colonic epithelium was observable by histological analysis (Fig. 6B and C), suggesting that streptomycin administration did not impact localization or composition of the mucus-associated microbiota.

## DISCUSSION

Here, we have demonstrated that microcontainers can be utilized for local delivery of antibiotics in the gut environment to selectively enhance the proliferation of an introduced resistant gut bacterium in the cecum and colon. We chose streptomycin as the model antibiotic, as this bactericidal drug is conventionally added to the drinking water at 5g/L to allow *in vivo* colonic establishment of orally introduced Gram-negative bacteria, such as E. coli (14–19). However, we found that this concentration of streptomycin also affected the small intestinal bacterial community and allowed E. coli engraftment also in this compartment. A 16S rRNA amplicon analysis revealed that Escherichia and *Shigella* constituted 6.72% (±7.87%) of the total bacterial community in the ileum of animals dosed with 5g/L of streptomycin in drinking water which, considering that E. coli is not endogenous to the ileum, appears high. Also, E. coli was observed to colonize the jejunum of animals only in the STR-W group (Fig. 3).

An important objective of our study was to compare the effect of delivering antibiotics contained in microcontainers as numerous small hot spots of high concentrations, which then diffuse into the local intestinal site, to that of a more constant and evenly distributed supply through the drinking water. While animals in the STR-W group were continuously exposed to streptomycin, animals receiving the drug in microcontainers were only exposed once every 24 h, which possibly created more fluctuating local intestinal concentrations. The significant E. coli decrease observed in fecal samples in the STR-M group between 24 and 48 h after inoculation, but not in the STR-W group, may be attributed to a decrease in the overall concentration of streptomycin delivered by microcontainers on the previous day, which did not occur in the animals that still had access to streptomycin-containing water 48 h after inoculation.

An unavoidable difference in the total amount of delivered streptomycin between the STR-W and STR-M groups is highly likely to play a role in our observations. Although the design was optimized to deliver as much streptomycin in microcontainers as possible, animals receiving streptomycin by this method were estimated to have received around 80-fold less streptomycin than those dosed with the standard of 5 g/L via drinking water. Nevertheless, the locally delivered lower dose of streptomycin significantly modulated the cecal and colonic microbiota and allowed engraftment of the introduced E. coli. Microcontainer-based delivery may thus be suited for interventions where a high local concentration but a low total use of antibiotics is desired.

To control streptomycin release from the microcontainers, we used the commercially available coating Eudragit S100, which is characterized as a colon-specific coating designed to dissolve above a pH of approximately 7 (20). The pH variation throughout the murine intestinal tract is less pronounced than in the human intestine (21), making pH-dependent targeted release more challenging. However, the pH has been reported to reach around 7 in the distal part of the rat ileum (22). Microcontainers coated with Eudragit S100 have previously been reported to release their content at pH 6.8 (23), indicating that distal ileal release occurs in rats. However, interindividual pH variations in the gut may explain that there were no detectable amounts of resistant E. coli in the gut of three rats receiving streptomycin in microcontainers, suggesting that no streptomycin was released from the drug delivery devices. Nevertheless, cecal, colonic, and fecal microbial communities from these three animals grouped with those from the other animals in the STR-M group, indicating that exposure to streptomycin had indeed occurred (Fig. 4C and 5C). We speculate that the release of streptomycin in these three rats failed on day 0 and that the intestinal environment was therefore not conditioned to allow proliferation of the E. coli, which was administered solely on day 1. Subsequently, release from microcontainers administered on day 2 could then have caused the observed changes in the endogenous microbiota but occurred at a time point when Str^r^ E. coli had already been cleared from the gut.

As E. coli is known to proliferate in murine colonic mucus (15), we sought to investigate the effect of this streptomycin delivery approach on the mucosal gut microbiota of the animals inoculated with Str^r^ E. coli. Despite the substantial E. coli increase observed by CFU counts in samples from the STR-W group 2 days after inoculation (Fig. 2B and 3), we did not observe Escherichia encroachment in the colonic mucosa by 16S amplicon sequencing of the mucus obtained by laser capture microdissection (LCM). We speculate that this orally added strain, which is foreign to the rat gut environment, only proliferated in the intestinal lumen and did not penetrate the inner mucus. Surprisingly, the significant gut microbiota changes induced by streptomycin in the STR-M and STR-W animals were not reflected in changes of the mucosal communities and did not affect the distance from the colonic epithelium to the gut microbiota. We did, however, observe a nonsignificant increase in the relative abundance in the mucosa of Akkermansia muciniphila, a gut commensal associated with a healthy mucus layer (24), in both of the groups receiving streptomycin. This is consistent with previous observations showing an increase in *A. muciniphila* colonization following broad-spectrum antibiotic treatment in humans (25).

### Conclusion.

Application of microcontainers, from which the site of delivery was controlled by an added enteric coating, allowed delivery of streptomycin locally in the gut in amounts sufficient for selective enhancement of introduced streptomycin-resistant E. coli in the cecum and colon of rats. The microcontainer delivery system did not affect the microbiota in jejunum and ileum, as opposed to the conventional delivery method of adding the antibiotic to the drinking water. This has implications for the use of microcontainer-delivered drugs in animal studies involving inoculation of resistant strains and in which only a local proliferation is desired. From the longer perspective, the application of microcontainers may open novel opportunities for antibiotic treatment of humans with fewer side effects on nontarget populations.

## MATERIALS AND METHODS

### Preparation of inoculum.

E. coli mCherry was derived from E. coli K-12 (MG1655) with a *lacIq-GenR-mCherry* gene chromosomally incorporated in the attTN7 site, which was kindly provided by Asmus K. Olesen, University of Copenhagen. We chose a fluorescence-expressing strain for easy visual verification. The genetically modified bacterium constitutively expresses fluorescent mCherry protein and is gentamicin resistant. For the study, a spontaneously streptomycin-resistant (MIC > 5 g/L) strain was isolated. The streptomycin-resistant E. coli mCherry was stored in LB medium with 15% glycerol (SSI Diagnostica A/S) at −80°C until use.

Before inoculation to rats, a Str^r^ colony was transferred to LB broth supplemented with 20 μg/mL gentamicin and 100 μg/mL streptomycin overnight or 16 h at 37°C with shaking. The culture was centrifuged at 5,000 × *g* for 10 min, washed, and resuspended in sterile phosphate-buffered saline (PBS) to a cell density of 10^9^ CFU/mL. An inoculum of 100 μL of this suspension, corresponding to 10^8^ CFU, was given to each rat.

### Fabrication, loading, and coating of microcontainers.

The microcontainers used in this study were kindly provided by Lasse Højlund Eklund Thamdrup, Department of Health Technology, Technical University of Denmark. They had a size of 250 μm (outer height) by 325 μm (outer diameter) and were fabricated from the negative epoxy photoresist SU-8 (SU-82035, 2075 and SU-8 developer; micro resist technology GmbH, Berlin, Germany) on Au-Ti-coated silicon substrates, through a previously described photolithography process (26).

The microcontainers were loaded with streptomycin by using polydimethylsiloxane (PDMS; Sylgard 184; Dow Corning, MI, USA) as previously reported (27). In short, PDMS (resin and cross-linker) was poured on the silicon substrates to mask the area between the microcontainers. After cross-linking of the PDMS, the streptomycin powder was compressed onto the reservoirs of the microcontainers using a spatula, after which the PDMS mask was manually peeled off using a tweezer.

The streptomycin-loaded microcontainers were sealed with the enteric coating Eudragit S100 (Evonik Industries, Darmstadt, Germany) as previously described (28). In brief, an isopropanol solution with 1% (wt/vol) Eudragit S100 and 0.5% (wt/vol) dibutyl sebacate (in relation to the polymer; Sigma-Aldrich, St. Louis, MO, USA) as plasticizer was prepared. The solution was spray coated at 40°C with a 2.2-W generator power and a 0.1-mL/min infusion rate, using a spray coater with an ultrasonic nozzle (ExactaCoat system; Sono-Tek, Milton, NY, USA). During the coating process, the nozzle speed was set at 10 mm/s, and the shaping air had 0.02 kPa pressure. The coated microcontainers were then kept at room temperature for up to 24 h for the coating to dry. For the *in vivo* experiment, coated microcontainers were manually scraped off the Au-Ti-coated silicon substrates and loaded into gelatin capsules (size 9; Torpac, Fairfield, NJ, USA). The final amount of streptomycin per loaded capsule was 3.19 mg (±0.44 mg). Capsules were stored at 5°C until use within a week.

### *In vivo* rat experiment.

Thirty Sprague-Dawley rats (Taconic Europe A/S), approximately 26 weeks old, were used to test the effect of streptomycin delivered by microcontainers on Str^r^ E. coli colonization and to evaluate the changes in microbiota composition. Before the experiment, all animals were singly housed and randomly allocated into three groups of 10 animals. Each animal was administered Str^r^ E. coli by oral gavage on day 1 and received a microcontainer-filled capsule at day 0, day 1, and day 2. The STR-M group received capsules containing streptomycin-filled microcontainers, whereas microcontainers received by the CTR group and STR-W group were empty. The STR-W group received streptomycin in the drinking water (5 g/L) from day 0. Animals had access to food (Altromin 1314) and water *ad libitum* before and during the experiment. Fresh fecal samples were collected directly from the individual animals before the start of the experiment on day 0 and on days 1, 2, and 3. On day 3, animals were sacrificed by CO_2_ asphyxiation followed by decapitation, and luminal samples from jejunum, distal ileum, cecum, and proximal colon were collected (Fig. 2A).

### CFU enumeration.

To quantify viable E. coli mCherry cells from feces and intestinal content during the *in vivo* study, CFU were counted. After collection, samples were serially diluted in PBS, plated on LB agar supplemented with 20 μg/mL gentamicin and 100 μg/mL streptomycin, and incubated at 37°C for 24 h before colonies were counted.

### Tissue processing for microscopy.

Immediately after euthanasia of the animals, 2-cm sections were collected from the jejunum, distal ileum, and proximal colon. Samples were placed in cassettes and fixated in methanol-Carnoy’s solution (60% methanol, 30% chloroform, and 10% acetic acid) for 48 h at room temperature. Tissues were deposited into a tissue processing device (Thermo Scientific Excelsior ES Tissue, Denmark) for ethanol dehydration serial steps (ethanol at 70%, 90%, 100%). After that, the tissues were embedded in paraffin and sectioned.

### Immunostaining and FISH.

To visualize bacteria and their localization in the tissue sections, immunostaining of the mucus was paired with fluorescent *in situ* hybridization (FISH) as modified by Johansson and Hansson (29). For confocal analyses, 8-mm sections were prepared and dewaxed at 60°C for 10 min, first with xylene for 10 min and then 99.5% ethanol for 5 min. Hybridization was performed at 50°C overnight with EUB338 probe (5′-GCTGCCTCCCGTAGGAGT-3′, with 5′ labeling using Alexa 647) diluted to a final concentration of 10 mg/mL in hybridization buffer (20 mM Tris-HCl [pH 7.4], 0.9 M NaCl, 0.1% SDS, 20% formamide). After washing 10 min in washing buffer (20 mM Tris-HCl [pH 7.4], 0.9 M NaCl) and a quick wash in PBS, slides were incubated in blocking solution (5% fetal bovine serum in PBS) in darkness at 4°C for 30 min. Slides were then gently dried and a PAP pen (Sigma-Aldrich) was used to encircle the section. Mucin-2 primary antibody (rabbit MUC2 antibody [C3], C-term; Genetex catalog number GTX100664) was diluted 1:100 in blocking solution and applied overnight at 4°C. After three 10-min washes in PBS, blocking solution containing anti-rabbit Alexa 488 secondary antibody (Invitrogen) diluted 1:300, phalloidin-tetramethylrhodamine B isothiocyanate (Sigma-Aldrich) at 1 mg/mL, and Hoechst 33258 (Sigma-Aldrich) at 10 mg/mL was applied to the section for 2 h. After three 10-min washes in PBS, slides were mounted using Prolong antifade mounting medium (Life Technologies) and kept in the dark at 4°C.

Samples for LCM were prepared as for confocal analyses with a few modifications. Arcturus PEN membrane frame slides (Applied Biosystems) were used and precoated with poly-l-lysine the day before by spreading 200 μL undiluted poly-l-lysine evenly on the membrane and dried overnight. In addition, samples were deparaffinized in xylene at room temperature, coverglasses were used instead of the PAP pen during incubation of primary MUC-2 antibody, and slides were not mounted with Prolong antifade medium. The slides were kept at 4°C until microdissection.

### Confocal laser scanning microscopy.

Images were acquired on a Spinning Disk IXplore using the Olympus cellSens imaging software (V2.3) at a frame size of 2,048 × 2,048 with 16-bit depth. A 405-nm laser was used to excite the Hoechst stain (epithelial DNA), 488 nm for Alexa Fluor 488 (mucus), 488 nm for tetramethyl rhodamine isocyanate (actin), and 640 nm for Alexa Fluor 647 (bacteria). Samples were imaged with a 20× objective or 60× oil immersion objective.

The epithelium-bacteria distance was calculated as follows: animals were randomized and 4 to 5 images showing intact mucus layers were selected from each animal. This step included five animals from the control group, four from the STR-M group, and nine from the STR-W group. For each image, five representative bacterial cells closest to the epithelium were selected, and the shortest distance was measured to the actin-stained epithelium border using the ImageJ software. Between 20 and 25 measurements were analyzed per animal.

### Laser capture microdissection.

Laser capture microdissection was performed as previously described (30). Section and microdissection were performed on an Arcturus laser capture microdissection system. Inner mucus layers from the samples were selected, as illustrated in Fig. 6C, and collected on CapSure Macro LCM caps (Arcturus) using a combination of infrared (IR) capture and UV (UV) laser cutting. A mean area of approximately 0.21 mm^2^ was collected from each sample. The membrane holding the inner mucus samples was carefully collected and stored at −80°C in DNA-free 0.5-mL tubes until DNA extraction.

### DNA isolation from the inner mucus layer.

The inner mucosal DNA was isolated using Qiagen QIAamp DNA Micro kit. In brief, 30 μL of ATL buffer and 20 μL of proteinase K were added to the microdissected samples and the mixtures were incubated at 56°C overnight. The next day, 50 μL of ATL buffer and 100 μL of AL buffer was added to each sample, followed by vortexing, and 100 μL of ethanol was added with a 5 min incubation at room temperature. The samples were transferred to a QIAamp MinElute column without the membrane and centrifuged at 6,000 × *g* for 1 min. The column was washed with 500 μL of AW1 buffer and 500 μL of AW2 buffer and dried by centrifugation at 20,000 × *g* for 3 min. Then, 20 μL of DNA-free water (Mobio) was added to the column center and the mixture was incubated at room temperature for 10 min, followed by centrifugation at 20,000 × *g* for 1 min to collect eluted DNA.

### DNA isolation, amplification, and 16S rRNA gene amplicon sequencing of fecal and intestinal contents.

Fecal and intestinal samples were treated as previously described (31).

### 16S rRNA amplicon analysis of fecal and luminal contents.

Analysis of the 16S rRNA gene amplicons was carried out essentially as previously described (31). In brief, sequences were demultiplexed and trimmed to remove barcodes and primers, and sequences below 125 bp or above 180 bp were discarded using the CLC Genomic Workbench (v8.5) software (CLCbio, Qiagen, Aarhus, Denmark). Using the DADA2 pipeline v1.12.44 in the R software (https://www.R-project.org/), reads were quality filtered (maxEE = 1, maxN = 0, truncQ = 2)and denoised (homopolymer gap penalty = −1, band size = 32), and chimeric sequences were removed. Amplicon sequence variants (ASVs) were assigned taxonomy using the RDP 16S rRNA database (rdp_train_set_18.fa.gz). Using QIIME2 (2019.1), ASVs with a frequency of <100 reads across all samples or assigned to Cyanobacteria/chloroplast were discarded. The core diversity metrics function was used with a rarefaction depth of 8,300 reads per sample to create Bray-Curtis distance matrices and alpha diversity measures (Shannon index). Using the rarefy table function, the ASV table was rarefied to 8,300 reads per sample and collapsed to genus levels using the taxa collapse function. Relative abundances at genus level were calculated by total sum scaling normalization.

### 16S rRNA amplicon sequencing and analysis of inner mucus microbiota.

Sequencing of 16S rRNA amplicons from inner mucus samples was performed as previously described using the Illumina MiSeq technology and following the protocol from Earth Microbiome Project (32).

Sequencing reads were demultiplexed and quality filtered using DADA2 (33) with QIIME2 default parameters. Taxonomy was assigned to the sequences with a 99% threshold of pairwise identity to the Greengenes reference database 13_8.

### Statistics.

Statistics were calculated using GraphPad Prism v. 9.0.2 unless otherwise stated. For comparisons of Str^r^ E. coli load, CFU counts were compared using multiple unpaired *t* tests between groups and paired *t* tests between days within groups. Alpha diversities were compared using the Wilcoxon matched-pairs signed-rank test for same-group comparisons and Kruskal-Wallis test for different group comparisons with adjusted *P* values (Dunn’s multiple-comparison test). The ANOSIM analyses were based on the Bray-Curtis dissimilarity matrix. All figures were generated using the GraphPad Prism software v 9.0.2 (GraphPad Software Inc., La Jolla, CA).

### Ethical approval.

Ethical approval was given by the Danish Animal Experiments Inspectorate (2015-15-0201-00553). The experiments were overseen by the National Food Institute’s inhouse Animal Welfare Committee for animal care and use.

### Data availability.

Sequence data have been deposited at the NCBI Sequence Read Archive under BioProject ID PRJNA764561.

## References

[1] Sommer F, Bäckhed F. 2013. The gut microbiota—masters of host development and physiology. Nat Rev Microbiol 11:227–238. 10.1038/nrmicro2974. 23435359

[2] Lawley TD, Walker AW. 2013. Intestinal colonization resistance. Immunology 138:1–11. 10.1111/j.1365-2567.2012.03616.x. 23240815 PMC3533696

[3] Ji SK, Yan H, Jiang T, Guo CY, Liu JJ, Dong SZ, Yang KL, Wang YJ, Cao ZJ, Li SL. 2017. Preparing the gut with antibiotics enhances gut microbiota reprogramming efficiency by promoting xenomicrobiota colonization. Front Microbiol 8:1208–1209. 10.3389/fmicb.2017.01208. 28702022 PMC5487471

[4] van der Waaij D, Berghuis-de Vries JM, Lekkerkerk-van der Wees JEC. 1971. Colonization resistance of the digestive tract in conventional and antibiotic-treated mice. J Hyg (Lond) 69:405–411. 10.1017/s0022172400021653. 4999450 PMC2130899

[5] Mazzoni C, Nielsen LH. 2020. Microdevices to successfully deliver orally administered drugs. *In* Martins JP, Santos HA (ed), Nanotechnology for Oral Drug Delivery. Elsevier, Philadelphia, PA.

[6] Feng K, Wei Y-s, Hu T-g, Linhardt RJ, Zong M-h, Wu H. 2020. Colon-targeted delivery systems for nutraceuticals: a review of current vehicles, evaluation methods and future prospects. Trends Food Sci Technol 102:203–222. 10.1016/j.tifs.2020.05.019.

[7] Nielsen L, Keller S, Boisen A. 2018. Microfabricated devices for oral drug delivery. Lab Chip 18:2348–2358. 10.1039/c8lc00408k. 29975383

[8] Mazzoni C, Jacobsen RD, Mortensen J, Jørgensen JR, Vaut L, Jacobsen J, Gundlach C, Müllertz A, Nielsen LH, Boisen A. 2019. Polymeric lids for microcontainers for oral protein delivery. Macromol Biosci 19:1900004. 10.1002/mabi.201900004. 30938933

[9] Christfort JF, Guillot AJ, Melero A, Thamdrup LHE, Garrigues TM, Boisen A, Zór K, Nielsen LH. 2020. Cubic microcontainers improve in situ colonic mucoadhesion and absorption of amoxicillin in rats. Pharmaceutics 12:355. 10.3390/pharmaceutics12040355. PMC7238233 32295139

[10] Nielsen LH, Melero A, Keller SS, Jacobsen J, Garrigues T, Rades T, Müllertz A, Boisen A. 2016. Polymeric microcontainers improve oral bioavailability of furosemide. Int J Pharm 504:98–109. 10.1016/j.ijpharm.2016.03.050. 27033999

[11] Dalskov Mosgaard M, Strindberg S, Abid Z, Singh Petersen R, Højlund Eklund Thamdrup L, Joukainen Andersen A, Sylvest Keller S, Müllertz A, Hagner Nielsen L, Boisen A. 2019. Ex vivo intestinal perfusion model for investigating mucoadhesion of microcontainers. Int J Pharm 570:118658. 10.1016/j.ijpharm.2019.118658. 31491485

[12] Conway T, Krogfelt KA, Cohen PS. 2004. The life of commensal Escherichia coli in the mammalian intestine. EcoSal Plus 10.1128/ecosalplus.8.3.1.2. 26443354

[13] Leatham-Jensen MP, Frimodt-Møller J, Adediran J, Mokszycki ME, Banner ME, Caughron JE, Krogfelt KA, Conway T, Cohen PS. 2012. The streptomycin-treated mouse intestine selects Escherichia coli envZ missense mutants that interact with dense and diverse intestinal microbiota. Infect Immun 80:1716–1727. 10.1128/IAI.06193-11. 22392928 PMC3347456

[14] Myhal ML, Laux DC, Cohen PS. 1982. Relative colonizing abilities of human fecal and K 12 strains of Escherichia coli in the large intestines of streptomycin-treated mice. Eur J Clin Microbiol 1:186–192. 10.1007/BF02019621. 6756909

[15] Poulsen LK, Lan F, Kristensen CS, Hobolth P, Molin S, Krogfelt KA. 1994. Spatial distribution of Escherichia coli in the mouse large intestine inferred from rRNA in situ hybridization. Infect Immun 62:5191–5194. 10.1128/iai.62.11.5191-5194.1994. 7927805 PMC303247

[16] Licht TR, Tolker-Nielsen T, Holmstrøm K, Krogfelt KA, Molin S. 1999. Inhibition of Escherichia coli precursor-16S rRNA processing by mouse intestinal contents. Environ Microbiol 1:23–32. 10.1046/j.1462-2920.1999.00001.x. 11207715

[17] Spees AM, Wangdi T, Lopez CA, Kingsbury DD, Xavier MN, Winter SE, Tsolis RM, Bäumler AJ. 2013. Streptomycin-induced inflammation enhances Escherichia coli gut colonization through nitrate respiration. mBio 4:430–443. 10.1128/mBio.00430-13. PMC3705454 23820397

[18] Adediran J, Leatham-Jensen MP, Mokszycki ME, Frimodt-Møller J, Krogfelt KA, Kazmierczak K, Kenney LJ, Conway T, Cohen PS. 2014. An Escherichia coli Nissle 1917 missense mutant colonizes the streptomycin-treated mouse intestine better than the wild type but is not a better probiotic. Infect Immun 82:670–682. 10.1128/IAI.01149-13. 24478082 PMC3911375

[19] Mokszycki ME, Leatham-Jensen M, Steffenson JL, Zhang Y, Krogfelt KA, Caldwell ME, Conway T, Cohen PS. 2018. A simple in vitro gut model for studying the interaction between Escherichia coli and the intestinal commensal microbiota in cecal mucus. Appl Environ Microbiol 84:e02166-18. 10.1128/AEM.02166-18. PMC6275339 30291119

[20] Zhang L, Cao F, Ding B, Li Q, Xi Y, Zhai G. 2011. Eudragit S100 coated calcium pectinate microspheres of curcumin for colon targeting. J Microencapsul 28:659–667. 10.3109/02652048.2011.604436. 21824069

[21] Merchant HA, Afonso-Pereira F, Rabbie SC, Youssef SA, Basit AW. 2015. Gastrointestinal characterisation and drug solubility determination in animals. J Pharm Pharmacol 67:630–639. 10.1111/jphp.12361. 25560785

[22] Afonso-Pereira F, Dou L, Trenfield SJ, Madla CM, Murdan S, Sousa J, Veiga F, Basit AW. 2018. Sex differences in the gastrointestinal tract of rats and the implications for oral drug delivery. Eur J Pharm Sci 115:339–344. 10.1016/j.ejps.2018.01.043. 29391214

[23] Nielsen LH, Rades T, Boyd B, Boisen A. 2017. Microcontainers as an oral delivery system for spray dried cubosomes containing ovalbumin. Eur J Pharm Biopharm 118:13–20. 10.1016/j.ejpb.2016.12.008. 27993733

[24] Dehghanbanadaki H, et al. 2020. Global scientific output trend for Akkermansia muciniphila research: a bibliometric and scientometric analysis. BMC Med Inform Decis Mak 20:1–12. 33167984 10.1186/s12911-020-01312-w PMC7654583

[25] Dubourg G, Lagier J-C, Armougom F, Robert C, Audoly G, Papazian L, Raoult D. 2013. High-level colonisation of the human gut by Verrucomicrobia following broad-spectrum antibiotic treatment. Int J Antimicrob Agents 41:149–155. 10.1016/j.ijantimicag.2012.10.012. 23294932

[26] Kamguyan K, Torp AM, Christfort JF, Guerra PR, Licht TR, Hagner Nielsen L, Zor K, Boisen A. 2021. Colon-specific delivery of bioactive agents using genipin-crosslinked chitosan coated microcontainers. ACS Appl Bio Mater 4:752–762. 10.1021/acsabm.0c01333.

[27] Kamguyan K, et al. 2020. Development and characterization of a PDMS-based masking method for microfabricated oral drug delivery devices. Biomed Microdevices 22:1–10. 10.1007/s10544-020-00490-8 32419094

[28] Abid Z, Strindberg S, Javed MM, Mazzoni C, Vaut L, Nielsen LH, Gundlach C, Petersen RS, Müllertz A, Boisen A, Keller SS. 2019. Biodegradable microcontainers-towards real life applications of microfabricated systems for oral drug delivery. Lab Chip 19:2905–2914. 10.1039/c9lc00527g. 31367713

[29] Johansson MEV, Hansson GC. 2012. Preservation of mucus in histological sections, immunostaining of mucins in fixed tissue, and localization of bacteria with FISH. Methods Mol Biol 842. 10.1007/978-1-61779-513-8_13 22259139

[30] Chassaing B, Gewirtz AT. 2019. Identification of inner mucus-associated bacteria by laser capture microdissection. Cell Mol Gastroenterol Hepatol 7:157–160. 10.1016/j.jcmgh.2018.09.009. 30510996 PMC6260373

[31] Laursen MF, Pekmez CT, Larsson MW, Lind MV, Yonemitsu C, Larnkjær A, Mølgaard C, Bode L, Dragsted LO, Michaelsen KF, Licht TR, Bahl MI. 2021. Maternal milk microbiota and oligosaccharides contribute to the infant gut microbiota assembly. ISME Commun 1:29–31. 10.1038/s43705-021-00021-3. PMC9723702 36737495

[32] Naimi S, Viennois E, Gewirtz AT, Chassaing B. 2021. Direct impact of commonly used dietary emulsifiers on human gut microbiota. Microbiome 9:1–19. 10.1186/s40168-020-00996-6. 33752754 PMC7986288

[33] Callahan BJ, McMurdie PJ, Rosen MJ, Han AW, Johnson AJA, Holmes SP. 2016. DADA2: high-resolution sample inference from Illumina amplicon data. Nat Methods 13:581–583. 10.1038/nmeth.3869. 27214047 PMC4927377

