# Supplementary material for: Local Delivery of Streptomycin in Microcontainers Facilitates Colonization of Streptomycin-Resistant Escherichia coli in the Rat Colon
Source: Appl Environ Microbiol. 2022 Jun 27;88(14):e00734-22. doi: 10.1128/aem.00734-22 (PMC9317935; doi:10.1128/aem.00734-22)
Supplement: Supplemental file 1 — Fig. S1. Download aem.00734-22-s0001.pdf, PDF file, 0.2 MB [file aem.00734-22-s0001.pdf]

Supplementary material

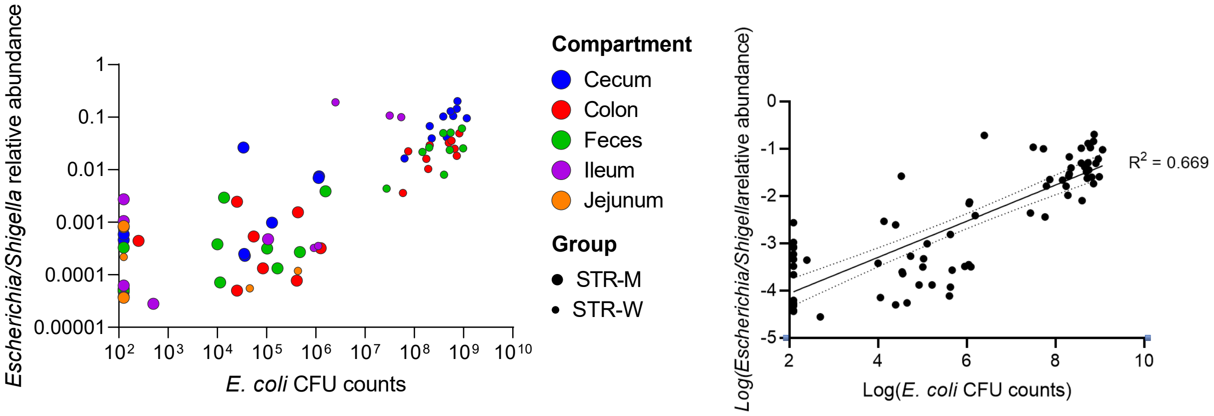

Supplementary Figure S1. Correlation between *Escherichia/Shigella* relative abundance from 16S rRNA amplicon sequencing and str^{R} *E. coli* CFU counts at Day 3.
